# Population Pharmacokinetics of Amikacin and Monte Carlo Simulation Study in Critically Ill Septic Patients Undergoing Continuous Renal Replacement Therapy with CytoSorb^®^ or oXiris^®^

**DOI:** 10.3390/pharmaceutics18101304

**Published:** 2026-10-14

**Authors:** Nikolina Špirić, Peđa Kovačević, Tijana Kovačević, Danica Momčićević, Saša Dragić, Biljana Zlojutro, Milka Jandrić, Goran Barić, Branislava Miljković, Marlies Ostermann, Katarina Vučićević

**Affiliations:** 1 Department of Pharmacokinetics and Clinical Pharmacy, Faculty of Pharmacy, University of Belgrade, 11221 Belgrade, Serbia; 2 Pharmacy Department, University Clinical Centre of the Republic of Srpska, 78000 Banja Luka, Bosnia and Herzegovina; 3 Medical Intensive Care Unit, University Clinical Centre of the Republic of Srpska, 78000 Banja Luka, Bosnia and Herzegovina; 4 Faculty of Medicine, University of Banja Luka, 78000 Banja Luka, Bosnia and Herzegovina; 5 Department of Critical Care, King’s College London, Guy’s and St Thomas’ NHS Foundation Trust, London SE1 9RT, UK

**Keywords:** amikacin, continuous renal replacement therapy, extracorporeal blood purification, population pharmacokinetics, Monte Carlo simulation, probability of target attainment, oXiris, CytoSorb, critically ill, therapeutic drug monitoring

## Abstract

**Background:** Adsorptive devices, including CytoSorb^®^ and oXiris^®^, are increasingly added to continuous renal replacement therapy (CRRT) in septic patients, but their effect on amikacin clearance and exposure is unknown. We characterized amikacin pharmacokinetics (PK) during adsorptive CRRT and derived dosing implications. **Methods:** A population PK model was developed in 24 critically ill septic patients (using NONMEM ver. 7.5.0), separating intrinsic clearance (CL_int) from filter-specific extracorporeal clearance (CL_CRRT). Sparse pre-filter sampling was handled using informative priors for distribution parameters. Robustness was assessed by sampling-importance-resampling (SIR) and prediction-corrected visual predictive checks (pcVPC). Monte Carlo simulations (9000 subjects) evaluated probability of target attainment (PTA; Cmax/MIC ≥ 8, AUC24/MIC ≥ 75) and cumulative fraction of response (CFR) against four Gram-negative pathogens across three regimens (500 mg q8h, 500 mg q24h, 1500 mg q24h), two filters, and three connection times. **Results:** While connected, the CytoSorb^®^ and oXiris^®^ CRRT configurations increased amikacin clearance by 61% and 93%, respectively, accounting for 38–48% of total clearance (CL_TOT). Because these devices were only applied for 10–14 h per 24 h interval in simulated scenarios, there were only minor differences in target attainment. Hence, dosing regimen, not device type or connection timing, dominated. Only amikacin at 1500 mg once daily achieved CFR ≥ 90%, and only for susceptible Enterobacterales. **Conclusions:** Adsorptive CRRT configurations substantially increase extracorporeal amikacin clearance. These exploratory findings support high-dose, once-daily, concentration-guided amikacin dosing during adsorptive CRRT, although this dose may be insufficient against pathogens with elevated MICs.

## 1. Introduction

Amikacin remains a valuable option for the short-term empirical or directed treatment of serious Gram-negative infections in critically ill patients with sepsis or septic shock. Among the commonly used aminoglycosides, it has a broader spectrum of activity than gentamicin and tobramycin. It is generally reserved for infections where drug resistance is suspected or confirmed, guided by local susceptibility patterns [1,2]. The bactericidal activity of amikacin is concentration-dependent. The Cmax/MIC ratio has traditionally been regarded as its principal pharmacokinetic/pharmacodynamic (PK/PD) efficacy index, with a target of approximately 8–10. AUC24/MIC has also been proposed as a complementary measure of antimicrobial exposure, which may be particularly relevant in critically ill patients with sepsis or septic shock because of their marked PK variability [2,3,4,5].

When interpreted in relation to contemporary European Committee on Antimicrobial Susceptibility Testing (EUCAST) breakpoints, these PK/PD targets highlight a major therapeutic challenge: conventional amikacin dosing may result in suboptimal or excessive exposure in critically ill patients with sepsis or septic shock. Systemic inflammation, capillary leak, fluid resuscitation, and evolving organ dysfunction can produce substantial and time-dependent changes in the volume of distribution and clearance of hydrophilic antimicrobials. Renal function may also change rapidly, ranging from augmented renal clearance to acute kidney injury (AKI) during the course of critical illness. Together with the narrow therapeutic index of aminoglycosides, this PK variability provides a strong rationale for therapeutic drug monitoring (TDM) and individualized dosing [6,7,8,9].

Continuous renal replacement therapy (CRRT), frequently used in hemodynamically unstable patients with severe AKI, introduces an additional and highly dynamic elimination pathway. Because amikacin has a low molecular weight (approximately 585 Da), is hydrophilic, and exhibits minimal protein binding, CRRT readily removes it. However, extracorporeal clearance is not fixed and may vary according to CRRT modality and intensity, effluent and blood flow rates, treatment duration and interruptions, residual renal function, and membrane characteristics. In addition to convective and diffusive clearance, adsorption to the filter membrane may contribute to amikacin removal, with its magnitude depending on membrane composition and surface properties [10,11,12,13,14,15,16]. Available PK evidence therefore supports an adequately high loading dose to achieve the desired peak concentration, followed by individualized redosing and guided by measured drug concentrations rather than fixed maintenance doses or intervals [10,11,12,13].

Extracorporeal blood purification, using adsorptive membranes or cartridges, is increasingly used as adjunctive therapy in severe septic shock. However, these devices may also remove concomitantly administered antimicrobials. Limited in vitro evidence indicates that polyacrylonitrile hemofilters can adsorb substantial amounts of amikacin, while broader experimental evidence demonstrates variable removal of anti-infective agents by dedicated hemoadsorption systems [17,18,19]. The clinical relevance of these effects remains uncertain and likely depends on membrane or device characteristics, treatment prescription and duration, and timing of antimicrobial administration [14]. However, direct clinical evidence comparing the effects of different adsorptive devices (CytoSorb^®^ vs. oXiris^®^) on amikacin disposition is lacking. It remains uncertain whether device-specific differences in amikacin removal result in clinically meaningful changes in extracorporeal clearance, systemic exposure, and PK/PD target attainment. This uncertainty is particularly relevant when hemoadsorption is applied for only part of the dosing interval, as a marked increase in clearance during device operation may not necessarily translate into a meaningful reduction in overall drug exposure. Population pharmacokinetic (PopPK) modelling can address these gaps by separating patient-related, non_CRRT clearance (CL_non_CRRT) from CRRT-associated clearance (CL_CRRT), thereby quantifying the additional extracorporeal clearance attributable to each device. Combined with Monte Carlo simulations, this approach can determine whether, and under which dosing regimens and device-connection timings, differences in CL_CRRT affect the probability of target attainment (PTA) and cumulative fraction of response (CFR). We therefore aimed to develop a PopPK model of amikacin in critically ill patients with sepsis or septic shock receiving CRRT with either a CytoSorb^®^ hemoadsorption cartridge or an oXiris^®^ adsorptive membrane, estimate configuration-specific CL_CRRT, and use Monte Carlo simulations to evaluate PTA and CFR across clinically relevant dosing regimens, device types, and connection timings.

## 2. Methods

### 2.1. Ethics and Trial Registration

This prospective observational study was conducted between 1 September 2023 and 31 December 2024 in the 28-bed Medical Intensive Care Unit of the University Clinical Centre of the Republic of Srpska, a tertiary university-affiliated hospital. The study protocol was approved by the institution’s Ethics Committee (approval no. 01-19-373-2/23, dated 27 September 2023). The study was conducted in accordance with the ethical principles outlined in the Declaration of Helsinki. Informed consent for participation was obtained from all patients involved in the study. For patients who were unable to provide informed consent due to impaired consciousness or cognitive impairment, written informed consent was obtained from their legally authorized representatives.

### 2.2. Patients

All consecutive adult patients (≥18 years) who met the Sepsis-3 criteria for sepsis or septic shock [20], developed sepsis-associated acute kidney injury (SA-AKI) requiring CRRT, and received treatment with either a CytoSorb^®^ hemoadsorption cartridge or an oXiris^®^ adsorptive membrane were eligible for inclusion. Hemoadsorption was initiated as adjunctive therapy in patients with severe systemic inflammation and evolving organ dysfunction, based on the clinical judgment of the treating intensivist; its use was not limited to patients requiring vasopressor support. All patients meeting the predefined eligibility criteria during the study period were prospectively screened and enrolled. The duration of recruitment was determined a priori on the basis of feasibility within routine clinical practice in a low-resource setting, including the anticipated availability of the hemoadsorption devices.

Patients were excluded if CRRT was initiated primarily for a condition unrelated to sepsis, including out-of-hospital cardiac arrest, rhabdomyolysis, intoxication, severe metabolic disturbances, AKI of non-septic aetiology, or acute heart failure with type 1 respiratory failure. Additional exclusion criteria were active malignancy and concomitant use of extracorporeal therapies that could introduce additional pathways of amikacin removal or sequestration, including the Molecular Adsorbent Recirculating System (MARS; Baxter International Inc., Deerfield, IL, USA), therapeutic plasma exchange, and extracorporeal membrane oxygenation (ECMO) or extracorporeal carbon dioxide removal using the PrisMax system (Baxter International Inc., Deerfield, IL, USA). Patients with a history of heparin-induced thrombocytopenia or heparin hypersensitivity were also excluded for safety-related reasons. All patients received standard treatment for sepsis or septic shock, including antimicrobial therapy, fluid resuscitation, vasopressor support when indicated, source control, and organ support, in accordance with institutional protocols and the Surviving Sepsis Campaign guidelines [21]. Patient demographic characteristics, clinical parameters, laboratory values, CRRT characteristics, and amikacin dosing information were collected prospectively. The following CRRT-related variables were recorded: CRRT modality, adsorptive device (CytoSorb^®^ or oXiris^®^), blood flow rate, dialysis rate, treatment duration, and fluid balance. In addition, relevant biochemical markers, namely serum creatinine (SCr), serum albumin (Alb), C-reactive protein (CRP) and interleukin-6 (IL-6), were obtained prior to and just after device disconnection.

### 2.3. Continuous Renal Replacement Therapy (CRRT) and Hemoadsorption

All patients with SA-AKI underwent continuous veno-venous hemodiafiltration (CVVHDF) using the Prismaflex system (Baxter International Inc., Deerfield, IL, USA). The prescribed effluent dose was 25–30 mL/kg/h to compensate for circuit downtime and treatment interruptions and to achieve a delivered dose of approximately 20–25 mL/kg/h, in accordance with guideline-based targets. Net fluid removal was individualized according to the patient’s hemodynamic status and did not exceed 2 mL/kg/h of ideal body weight. Anticoagulation with either regional citrate or systemic heparin was prescribed according to clinical and laboratory parameters, including platelet count, coagulation status, and contraindications to the respective anticoagulant. Hemoadsorption was initiated after initial resuscitation and hemodynamic stabilization, including fluid resuscitation and vasopressor support when indicated. The timing of initiation was determined by the treating intensivist according to the patient’s clinical condition and was not influenced by device availability.

Two distinct extracorporeal circuit configurations were used. In the CytoSorb^®^ group, CVVHDF was performed using exclusively the Prismaflex CRRT ST150 set (Baxter International Inc., Deerfield, IL, USA). The ST150 set contains an AN69ST hollow-fiber hemofilter composed of acrylonitrile and sodium methallyl sulfonate copolymer, surface-treated with polyethyleneimine. The CRRT hemofilter has an effective membrane surface area of 1.5 m^2^, an internal fiber diameter of 240 μm, and a fiber wall thickness of 50 μm. The nominal blood volume of the complete ST150 set is 193 mL.

The CytoSorb^®^ cartridge (CytoSorbents Corporation, Princeton, NJ, USA) is a sterile, single-use hemoadsorption device containing highly porous divinylbenzene copolymer beads. It is designed to adsorb a broad range of predominantly hydrophobic inflammatory mediators and cytokines through size-dependent capture and hydrophobic interactions. In the CytoSorb^®^ group, the cartridge was incorporated in series into the extracorporeal circuit downstream of the ST150 CRRT hemofilter, in the post-filter/post-dialyzer venous return line. Thus, blood first passed through the ST150 hemofilter and subsequently through the CytoSorb^®^ cartridge before returning to the patient. The extracorporeal blood volume of the CytoSorb^®^ cartridge is approximately 150 mL. The cartridge was connected directly to the standard Prismaflex circuit using an additional tubing segment with an approximate blood volume of 50 mL. Accordingly, the total nominal extracorporeal blood volume of the CytoSorb-containing circuit was approximately 393 mL. Each CytoSorb^®^ cartridge was used for 12–24 h and was then removed [22,23].

In the oXiris^®^ group, CVVHDF was performed using the oXiris^®^ set (Baxter International Inc., Deerfield, IL, USA), which served simultaneously as the CRRT hemofilter and the adsorptive membrane; no additional hemoadsorption cartridge was incorporated into the circuit. The oXiris^®^ hemofilter consists of an AN69-based membrane composed of acrylonitrile and sodium methallyl sulfonate copolymer fibers, surface-treated with polyethyleneimine and pre-grafted with heparin (approximately 4500 ± 1500 IU/m^2^). The membrane combines renal replacement therapy with endotoxin and cytokine adsorption and has an effective surface area of 1.5 m^2^, an internal fiber diameter of 240 μm, and a wall thickness of 50 μm [24,25,26]. The nominal blood volume of the complete oXiris^®^ set is 193 mL. oXiris^®^ treatment was continued for up to 24 h. CytoSorb^®^ and oXiris^®^ were never used simultaneously within the same extracorporeal circuit, nor were they used sequentially in the same patient during the study period. Therefore, the two treatment groups represented distinct extracorporeal configurations: ST150-based CVVHDF with an additional post-filter CytoSorb^®^ cartridge vs. oXiris^®^-based CVVHDF without an additional hemoadsorption cartridge.

For both treatment configurations, the start and end times of each hemoadsorption session and the total treatment duration were prospectively recorded. The choice between the CytoSorb^®^-containing and oXiris^®^-based circuit configurations was made by the treating intensivist as part of routine clinical care. oXiris^®^ was preferentially selected when Gram-negative sepsis and associated endotoxemia were clinically suspected, particularly in patients with presumed nosocomial infection, based on the available clinical and microbiological findings. Because direct endotoxin measurement was unavailable, endotoxin concentrations or endotoxin activity assays were not used to guide device selection. All patients received empirical antimicrobial therapy based on the suspected source of infection, previous microbiological findings, and local resistance patterns. Microbiological samples were obtained before initiation of empirical antimicrobial therapy whenever this was feasible and did not delay treatment.

### 2.4. Amikacin Dosing and Pharmacokinetic Sampling

Amikacin was administered at 500 mg as a 0.5 h short infusion, at 8, 12, or 24 h dosing intervals according to the clinician’s judgement and the patient’s renal function. A single blood sample was obtained immediately before filter connection for each patient. Multiple blood samples (typically 4–7 per patient, at: 15 min, 30 min, 1 h, 3 h, 6 h, and immediately before the next dose depending on dosing interval) were obtained over the course of the filter-active period. Sampling times were recorded both relative to the most recent dose (time after dose) and relative to filter connection (time since filter). No further samples for PK analyses were collected after filter disconnection. Device type (CytoSorb^®^, oXiris^®^, or none) and its length of treatment were recorded at every sampling time. Serum amikacin concentrations were measured using a validated Roche Cobas enzyme immunoassay method (Roche Diagnostics GmbH, Mannheim, Germany) with a lower limit of quantification (LLOQ) of 1.2 μg/mL [27].

### 2.5. Population Pharmacokinetic Analysis

PopPK analysis was performed using nonlinear mixed-effects modeling in NONMEM^®^ (version 7.5.0, ICON Development Solutions, Ellicott City, MD, USA), executed with the first-order conditional estimation method with interaction (FOCE-I) [28]. Based on raw data, a two-compartment disposition model was fitted using the general nonlinear differential-equation solver (ADVAN13/$DES), with elimination from the central compartment. Because the sampling design provided only a single pre-filter concentration per patient, the intercompartmental clearance (Q) and peripheral volume of distribution (V2) could not reliably be estimated from the present data alone. These parameters were, therefore, constrained using informative priors implemented via the NONMEM PRIOR subroutine (NWPRI, a normal-inverse-Wishart prior on THETA/OMEGA), following published methodological guidance [29]. Prior means and variances were taken from a published two-compartment PopPK model of amikacin developed from rich sampling scheme specifically for Bayesian estimation in critically ill septic patients [30], selected for its congruence with our population, identical structural model, and explicit characterization of the distributional parameters. CL_int and central volume of distribution (V1) were estimated freely, with exponential interindividual variability (IIV) on both parameters using a full covariance structure. Residual unexplained variability was described using a combined error model. CL_CRRT was modelled as an additive, filter-type-specific term CL_CRRT(CytoSorb) or CL_CRRT(oXiris), applied only while the corresponding device was in use. In the base model, this term was estimated at the population level: CL_TOT=CL_int+CL_CRRT. Two model extensions were tested as well: (i) addition of IIV on CL_CRRT, to test whether CL_CRRT varies meaningfully between individuals; (ii) a step increase in V1 at the moment of device connection. This was implemented using the existing time-varying filter indicator to test whether a discrete change in apparent volume at circuit connection could account for the residual variability localized to the pre-filter and transition samples. Potential covariates (including body weight, renal function markers, length of adsorptive CRRT, blood flow, rate of dialysis, and clinical biomarkers) affecting structural parameters were evaluated via the stepwise-covariate-modeling (SCM) approach with the forward criteria of ΔOFV ≥ 3.84 (df = 1, α = 0.05) and ΔOFV ≥ 6.63 (df = 1, α = 0.01) for backward step.

### 2.6. Model Evaluation and Sensitivity Analyses

Model discrimination used standard criteria: the change in objective function value (ΔOFV, likelihood ratio test, requiring ΔOFV ≥ 3.84 for one additional parameter at α = 0.05), successful covariance step, condition number, precision of parameter estimates (value of relative standard error, RSE, from the covariance step), η- and ε-shrinkage, and biological plausibility. Both extensions previously mentioned were evaluated against the ΔOFV and RSE criteria. Moreover, standard graphical diagnostics were used as well. They included the following plots: observed (DV) vs. population/individual predictions (PRED/IPRED); conditional weighted residuals (CWRES) vs. time/PRED [28]. As the sampling density differed markedly between the pre-extracorporeal phase (single point per patient) and the within-extracorporeal phase (rich sampling), residual diagnostics addressed phase- and filter-associated data to assess whether goodness-of-fit (GOF) was uniform across the sampling design. In addition, prediction-corrected visual predictive checks (pcVPC) and sampling importance resampling (SIR)-based confidence intervals (CIs) for the final parameters were performed for the final model.

### 2.7. Monte Carlo Simulation and PK/PD Target Attainment Analysis

To translate the final PopPK model into dosing guidance, Monte Carlo simulations were performed using the final model parameter estimates, implemented in NONMEM^®^ (version 7.5.0, ICON Development Solutions, Ellicott City, MD, USA) as a simulation-only control stream [28]. Eighteen scenarios were simulated, corresponding to the full factorial combination of three amikacin dosing regimens: 500 mg q8h, 500 mg q24h and 1500 mg q24h for 3 days, two extracorporeal modalities (CRRT with CytoSorb, duration 10 h and CRRT with oXiris, duration 14 h), and three filter-initiation times anchored to the second day of therapy: 2 h before the dose, 1 h after the dose, and at half the dosing interval after the dose. For each scenario, 500 virtual patients were simulated, and IIV on CL and V1 were resampled from the estimated variance-covariance structure, and concentrations were generated on a 5 min time grid over 96 h. Adsorptive CRRT status was encoded as a time-varying covariate with paired change-point records at the exact moment of device connection and disconnection, ensuring the model-predicted step change in CL_TOT precisely at the intended time regardless of the surrounding observation grid. Exposure metrics (Cmax, and AUC24 using the trapezoidal method) were calculated for each simulated patient from the model-predicted (noise-free) concentration profile during the second day of therapy. Cmax/MIC and AUC24/MIC ratios were calculated across an MIC range of 0.002 to 512 mg/L. The PTA at each MIC was defined as the percentage of simulated patients achieving conventional primary Cmax/MIC ≥ 8 or secondary target AUC24/MIC ≥ 75. The CFR for each scenario was calculated by weighting the PTA at each MIC by the corresponding probability mass of that MIC in the EUCAST MIC distribution for four clinically relevant Gram-negative pathogens (*Acinetobacter baumannii*, *Escherichia coli*, *Klebsiella pneumoniae*, *Pseudomonas aeruginosa*). A CFR ≥ 90% was considered indicative of optimal probability of achieving the PK/PD target. Dataset preparation, post-processing of outputs, and graphical presentation of results were performed using R software (version 4.6.0; R Foundation for Statistical Computing, Vienna, Austria) within RStudio Desktop (version 2025.09.0; Posit Software, PBC, Boston, MA, USA), with various R packages.

## 3. Results

### 3.1. Patients and Dataset

Twenty-four patients contributed 156 quantifiable amikacin concentrations: 22 obtained prior to device connection, 61 during CytoSorb^®^, and 73 during oXiris^®^ therapy (Table 1). Baseline renal function was markedly heterogeneous, consistent with the co-occurrence of AKI and augmented clearance within this population prior to filter initiation. Eleven patients were treated with CytoSorb^®^ and thirteen received oXiris^®^ hemoadsorption.

CRRT characteristics were broadly comparable between groups, although the modest sample size limits the power of these comparisons. There was a non-significant trend toward higher blood flow rates in the CytoSorb^®^ group (median 120 vs. 100 mL/min; *p* = 0.095), and median dialysate flow was 1200 mL/h in both groups (*p* = 0.208). No significant differences were observed in extracorporeal treatment duration (median 16.0 vs. 21.75 h; *p* = 0.311) and fluid balance (median 4.5 vs. 5.25 L; *p* = 0.376). Baseline renal function and inflammatory status were also similar, with no significant between-group differences in serum creatinine (median 301 vs. 265 µmol/L; *p* = 0.26). All comparisons used the Mann–Whitney U test.

### 3.2. Final Population Pharmacokinetic Model

A two-compartment model with additive, filter-type-specific CL_CRRT provided a stable, well-conditioned fit (minimization successful, condition number ≈ 22). SCM was performed to evaluate the effects of the prespecified covariates on PK parameters. None of the investigated covariates met the predefined significance criteria and they had no significant effect on CL, V1, or CL_CRRT. Parameter estimates were obtained with acceptable precision (RSE 8.7–26.8%). Q and V2 were informed by the prior and estimated at 4.70 L/h and 9.15 L, respectively, consistent with the prior means. The correlations between these parameters and the clearance estimates were negligible in both the covariance step (|r| ≤ 0.12) and the SIR analysis (|r| ≤ 0.09), indicating that the priors had minimal influence on the estimated intrinsic and extracorporeal clearances. During device connection, extracorporeal clearance was estimated at 1.70 L/h for the CytoSorb^®^ configuration and 2.58 L/h for the oXiris^®^ configuration, corresponding to increases of 61% and 93% over CL_int, respectively (Table 2). These configuration-specific estimates are descriptive and, given non-randomized allocation, do not support a direct comparison between devices. η-shrinkage was low for both random effects, and residual variability was well described overall (population-average CWRES standard deviation ≈ 0.99). Final model parameter estimates are summarized in Table 2.

SIR was used to quantify parameter uncertainty: SIR medians were close to the point estimates for all parameters, and the 95% CIs for both configuration-specific CL_CRRT terms excluded zero, indicating non-negligible extracorporeal clearance with each configuration (Table 2). The wider relative interval for CL_int and its high IIV reflect the pronounced PK heterogeneity expected in this critically ill population.

**Figure 1:**
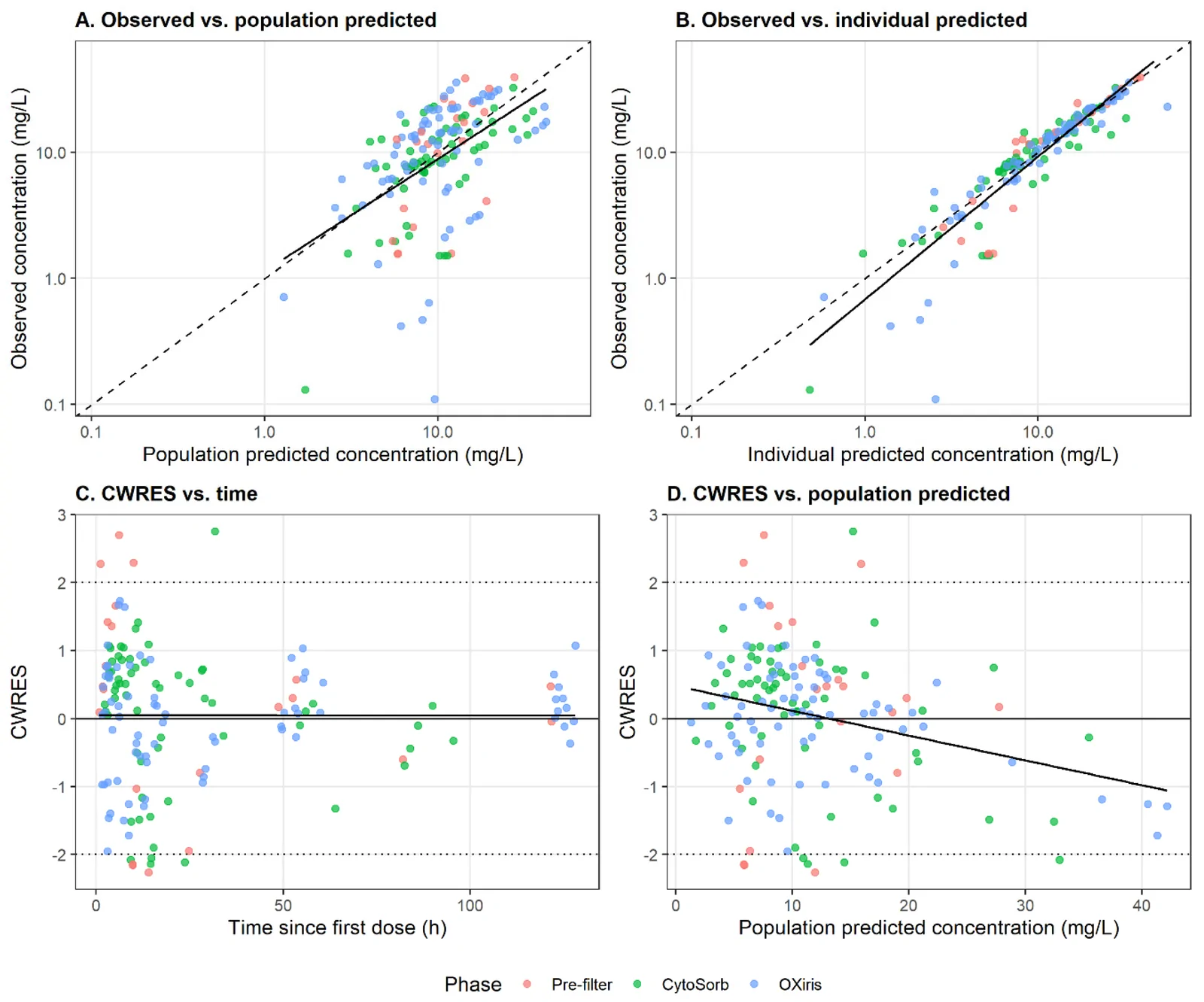
Goodness-of-fit and phase-stratified residual diagnostics for the final amikacin population pharmacokinetic (PK) model. (**A**) Observed vs. population-predicted (PRED) and (**B**) observed vs. individual-predicted (IPRED) amikacin concentrations; the dashed line is the line of identity and the solid line a fitted trend. (**C**) Conditional weighted residuals (CWRES) vs. time since first dose and (**D**) CWRES vs. PRED; the solid horizontal line marks CWRES = 0 and the dotted lines the ±2 reference interval. In all panels, points are colored by sampling phase (pre-filter, CytoSorb^®^, oXiris^®^).

GOFs (Figure 1) supported the adequacy of the final model. Observed concentrations were symmetrically distributed around the line of identity for both PRED and IPRED (Figure 1A,B), and CWRESs were evenly scattered around zero without systematic trend across time after dose or PRED, with the large majority falling within the ±2 reference interval (Figure 1C,D). Residual dispersion was greater at the single pre-filter sample per patient (CWRES standard deviation 1.47) than during the densely sampled CytoSorb^®^ and oXiris^®^ phases (1.01 and 0.80, respectively), consistent with the limited information contributed by the sparse pre-filter sampling. Inspection of the raw concentration–time data showed that this transition-phase misfit corresponded to a rapid fall in amikacin concentration around device connection: among patients with no dose administered between the last pre-device and first on-device sample, concentrations declined by a median of 21% (range 3–58%) within approximately 30–45 min of filter connection, substantially greater than the ~10% decline predicted by elimination alone over the same interval.

**Figure 2:**
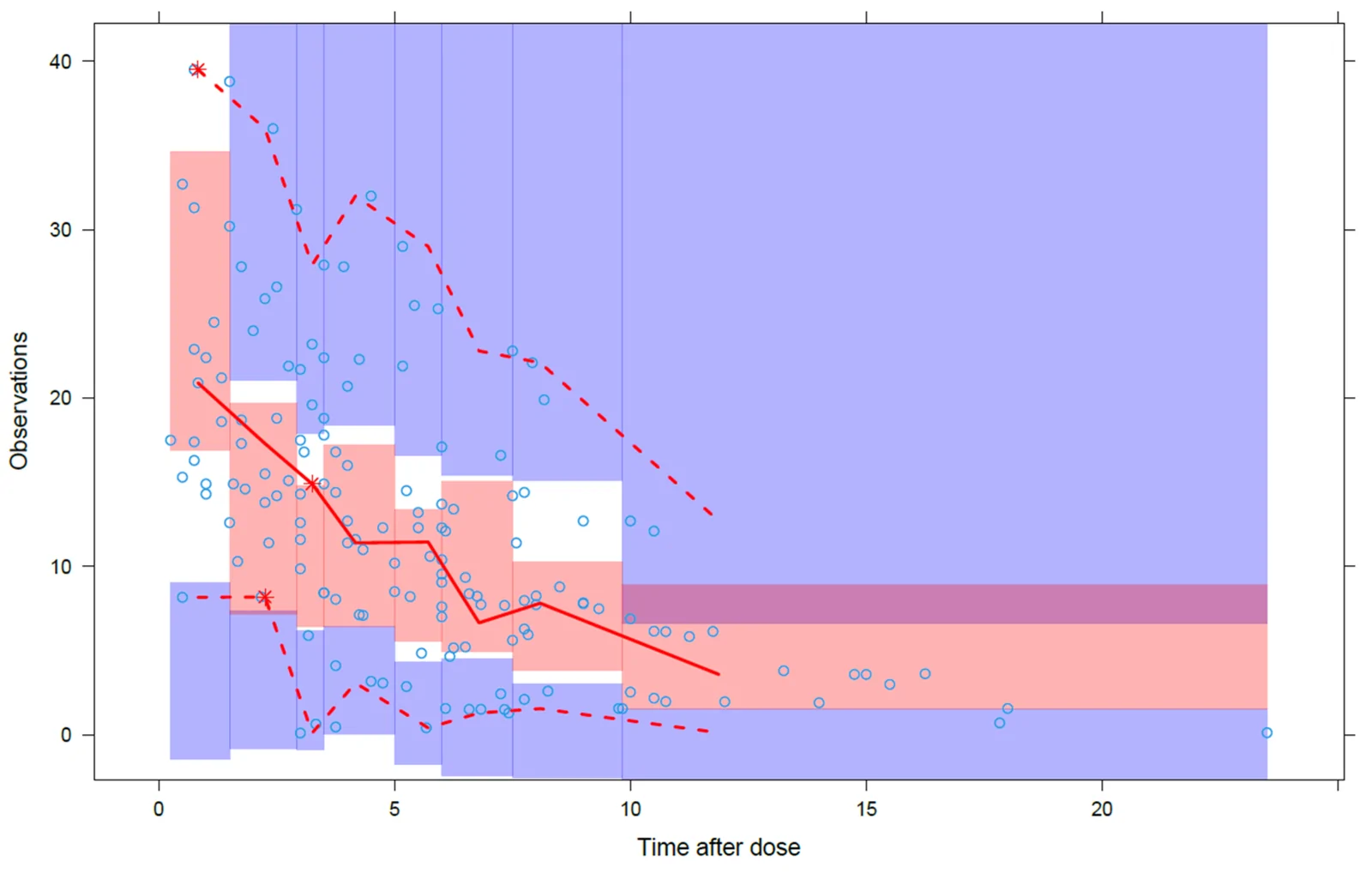
Prediction-corrected visual predictive check (pcVPC) of the final amikacin population pharmacokinetic model. Observed amikacin concentrations (open circles) are plotted against time after dose. Solid and dashed lines represent the median and the 5th and 95th percentiles of the observed data, respectively. Red and blue shaded areas represent the 95% confidence intervals of the model-predicted median and of the 5th and 95th percentiles, respectively, obtained from 500 simulated replicates of the original dataset.

The pcVPC showed that the model adequately described the central tendency and variability of the observed concentrations across the sampled time course (Figure 2).

### 3.3. Sensitivity Analyses

Two pre-specified structural extensions, aimed at explaining the elevated residual variability at the pre-device and transition samples, were evaluated and neither was retained.

Adding IIV to CL_CRRT produced near-complete η-shrinkage (96%) and degraded the precision of the device-specific clearance estimates (RSE increasing from 21–27% to 45%), with no meaningful reduction in OFV. This is consistent with the identifiability limit imposed by the sampling design: a single pre-device concentration combined with one continuous filter run per patient permits estimation of CL_TOT during CRRT at the individual level, but not its decomposition into intrinsic and extracorporeal components. CL_CRRT was therefore retained as a population-level, device-type-specific parameter.

Second, a step increase in V1 at the moment of device connection was tested against the transient concentration decline observed at that time. It did not improve the fit (ΔOFV = 1.27, df = 1, *p* > 0.05), was imprecisely estimated (RSE = 89%), and did not resolve the elevated transition-phase residual variability. The transient peri-connection decline was therefore not incorporated as a model feature.

### 3.4. Monte Carlo Simulation: PK/PD Target Attainment and Cumulative Fraction of Response

Across the 18 simulated scenarios and 9000 simulated individuals in total, day-2 exposure differed markedly between regimens. Median Cmax was highest for 1500 mg q24h (76.0 mg/L), intermediate for 500 mg q8h (35.7 mg/L), and lowest for 500 mg q24h (24.9 mg/L). In contrast, median AUC24 was near-identical for 1500 mg q24h and 500 mg q8h (392 and 404 mg·h/L, respectively) and roughly threefold lower for 500 mg q24h (128 mg·h/L). Thus, the once-daily high-dose regimen maximized Cmax-driven exposure, while the two 1500 mg/day regimens were equivalent in cumulative exposure.

**Figure 3:**
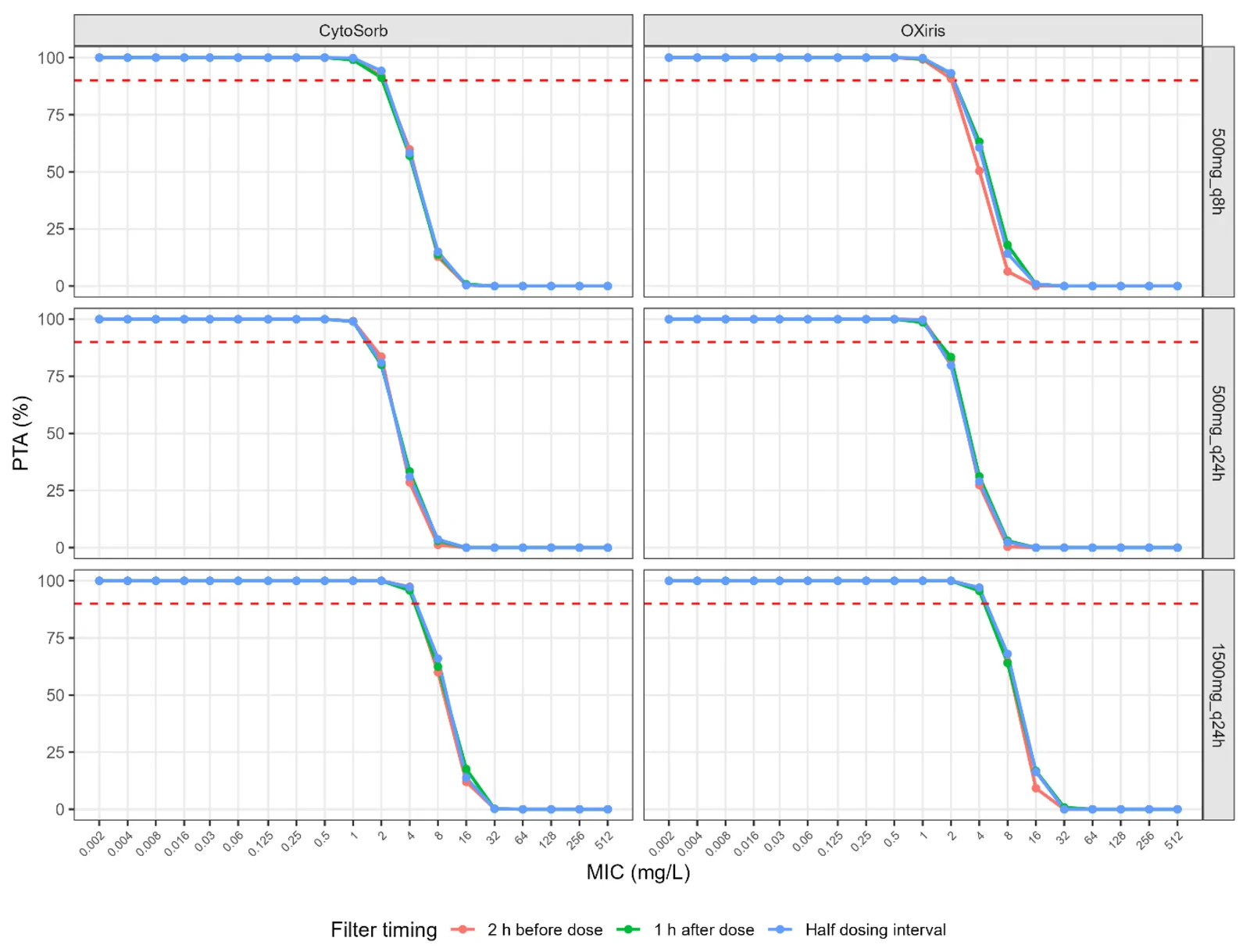
Probability of target attainment (PTA) for Cmax/MIC ≥ 8 vs. minimal inhibitory concentration (MIC) for amikacin dosing regimens, filter type and length. Dashed red line represents 90% PTA.

**Figure 4:**
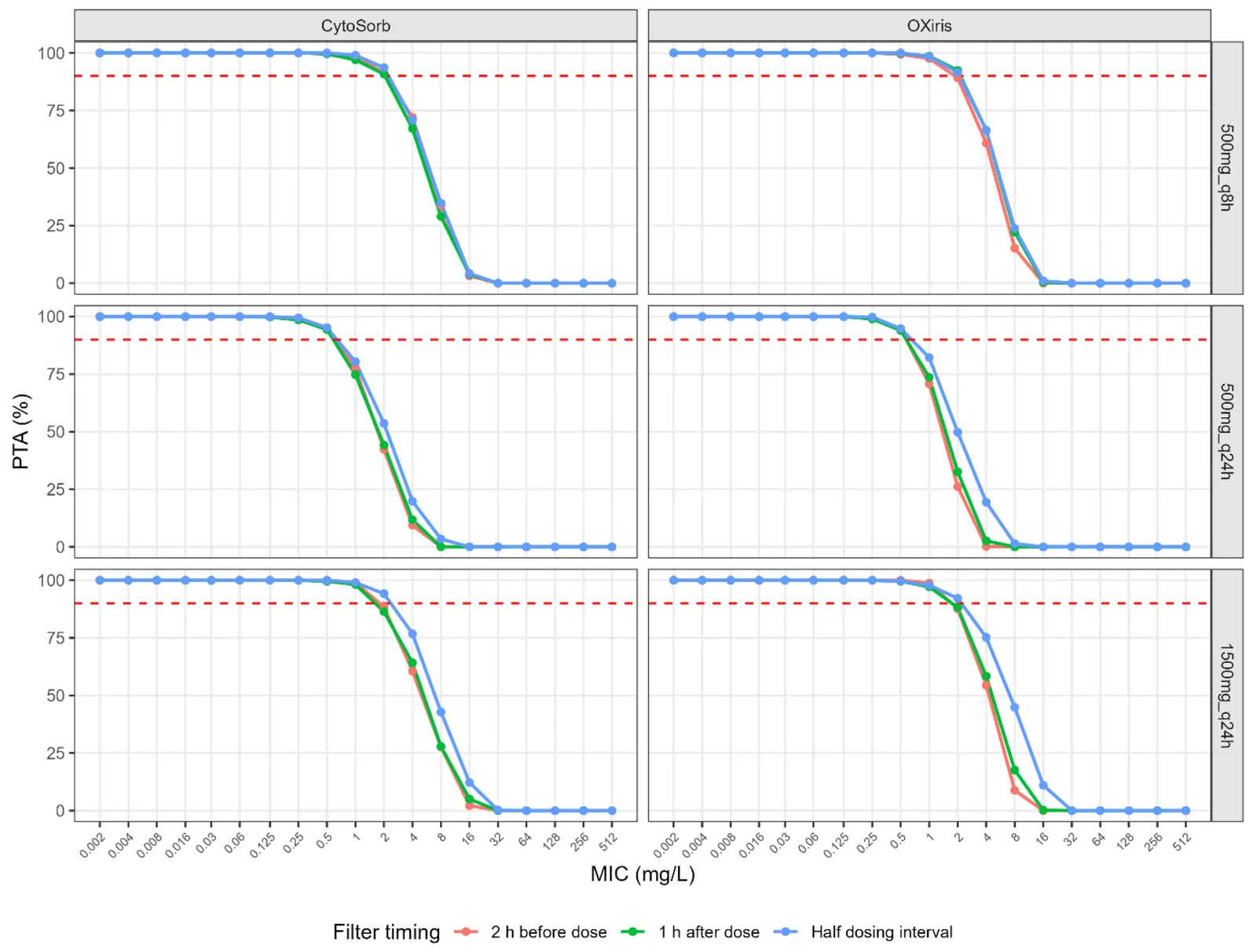
Probability of target attainment (PTA) for AUC24/MIC ≥ 75 vs. minimal inhibitory concentration (MIC) for amikacin dosing regimens, filter type and length. Dashed red line represents 90% PTA.

PTA declined with increasing MIC in all scenarios (Figure 3 and Figure 4). For the Cmax/MIC ≥ 8 target (Figure 3), PTA remained ≥90% up to an MIC of 2 mg/L for the 500 mg q8h and 1500 mg q24h regimens but fell below 90% at 1–2 mg/L for 500 mg q24h. At an MIC of 4 mg/L, PTA was approximately 50–65% for the higher-exposure regimens and below 35% for 500 mg q24h, and was negligible (<5%) at MICs ≥ 16 mg/L across all regimens. For the AUC24/MIC ≥ 75 target (Figure 4), 500 mg q8h and 1500 mg q24h again retained ≥90% PTA up to an MIC of 2 mg/L, whereas 500 mg q24h fell below 90% at an MIC of 0.5–1 mg/L.

Two features were consistent across both targets. First, filter-connection timing had minimal influence on target attainment: the PTA–MIC curves for the three timing offsets were near-superimposable in every scenario. Second, the difference between CytoSorb^®^ and oXiris^®^ was small despite the higher CL_CRRT estimated with oXiris^®^ treatment. PTA differed by only a few percentage points between devices, with marginally lower attainment under oXiris^®^ at intermediate MICs.

CFR was consistently highest against *Escherichia coli* and *Klebsiella pneumoniae* and lowest against *Acinetobacter baumannii* and *Pseudomonas aeruginosa*, reflecting the left-shifted MIC distributions of the former and the broader distributions of the latter. Amikacin dose was the dominant determinant, as the 500 mg q24h regimen performed worst for every pathogen, device, and timing combination, while filter type and timing again had little effect (CFR differences were generally ≤5%).

**Figure 5:**
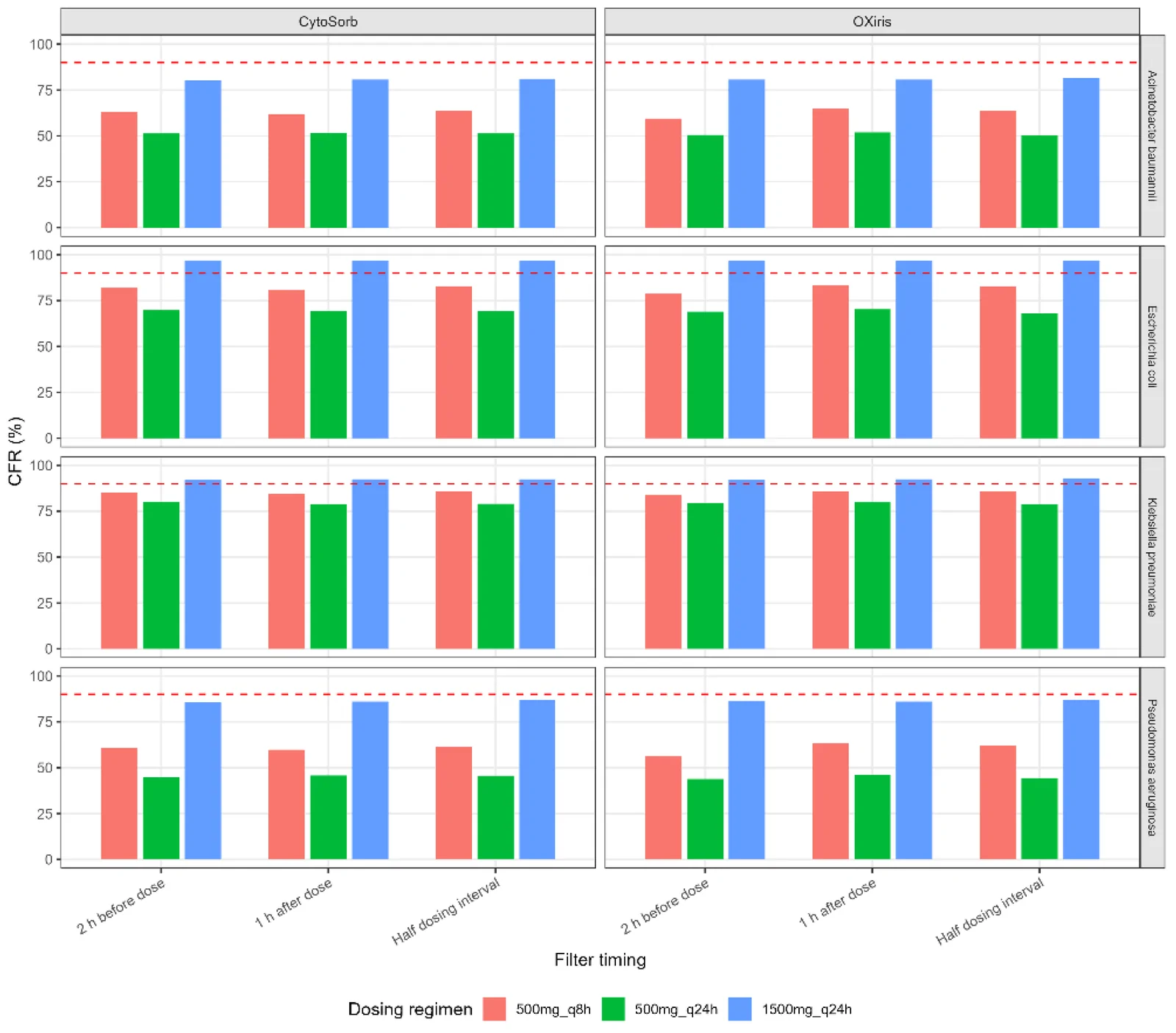
Cumulative fraction of responses (CFRs) vs. amikacin dosing regimens, filter type and length stratified by four pathogens and Cmax/MIC ≥ 8 as PK/PD target. Dashed red line represents 90% CFR.

**Figure 6:**
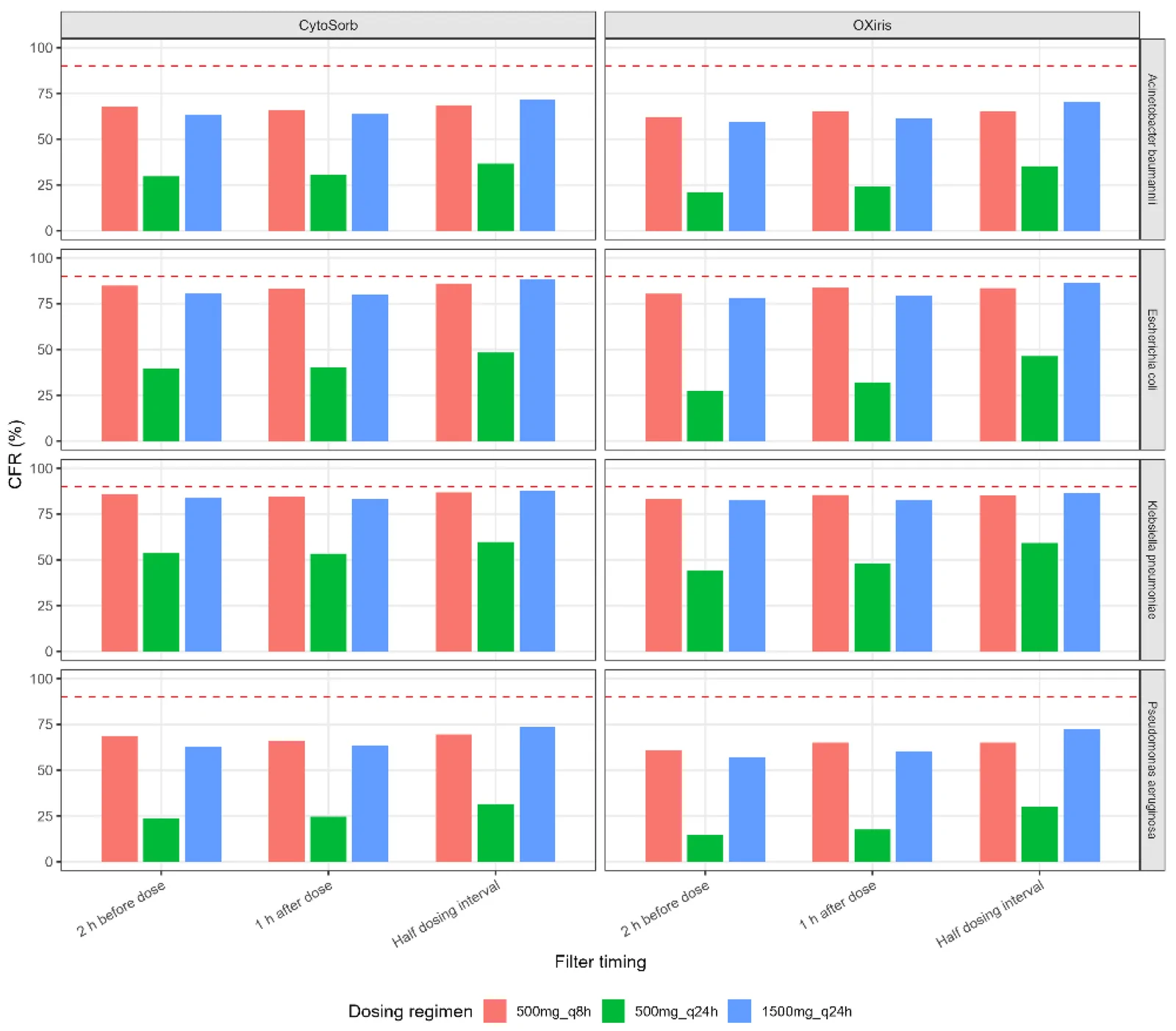
Cumulative fraction of responses (CFRs) vs. amikacin dosing regimens, filter type and length stratified by four pathogens and AUC24/MIC ≥ 75 as PK/PD target. Dashed red line represents 90% CFR.

For the Cmax/MIC ≥ 8 target, the 1500 mg q24h regimen achieved the highest CFR of 92–93% against *K. pneumoniae*, ~97% against *E. coli*, ~86–87% against *P. aeruginosa*, and ~80–81% against *A. baumannii* (Figure 5). The 500 mg q8h regimen reached ~85% CFR against *K. pneumoniae* and ~80–83% against *E. coli* but only ~60–65% against *A. baumannii* and *P. aeruginosa*, and 500 mg q24h fell to ~50–52% and ~44–46% against these two non-fermenters, respectively (Figure 5). For the more stringent AUC24/MIC ≥ 75 target, no regimen reached 90% CFR against any pathogen (Figure 6). The best CFR values were ~86–88% (1500 mg q24h and 500 mg q8h against *E. coli* and *K. pneumoniae*), while CFR against *A. baumannii* and *P. aeruginosa* remained below ~75% under all scenarios and fell as low as ~15–30% for 500 mg q24h (Figure 6).

Overall, only the 1500 mg q24h regimen provided adequate (≥90%) target attainment, and only against the more susceptible Enterobacterales (*E. coli*, *K. pneumoniae*) for the peak-based target. No simulated regimen achieved satisfactory CFR against *A. baumannii* or *P. aeruginosa*, regardless of device type or connection timing.

## 4. Discussion

In our study, we characterized PopPK of amikacin in critically ill septic patients transitioning onto CRRT combined with an adsorptive device (CytoSorb^®^ or oXiris^®^), using a two-compartment model that separates CL_int from configuration-specific extracorporeal clearance. Two findings anchor this work. First, both adsorptive CRRT configurations added a substantial increment to amikacin CL while connected, estimated at 1.70 L/h (95% CI 0.86–2.57) for the CytoSorb^®^ configuration and 2.58 L/h (1.67–3.69) for the oXiris^®^ configuration, corresponding to 61% and 93% of CL_int, respectively. At typical parameter values, this shortens the half-life of elimination from the central compartment from 4.8 h to 3.0 h and 2.5 h, respectively, and the extracorporeal component accounted for 38% and 48% of CL_TOT during device therapy. Given the non-randomized allocation and overlapping confidence intervals, these estimates describe each configuration and do not support a direct comparison between devices.

The effect of hemoadsorption on antimicrobial drug disposition remains insufficiently characterized, particularly in critically ill patients receiving concurrent CRRT. In vitro studies reported an initial 4.6–14.4% reduction in amikacin concentrations during the first 15 min of hemoadsorbent exposure, followed by substantially slower removal, suggesting early saturation of adsorption capacity [31]. Limited clinical data from pediatric patients receiving hemoadsorption combined with CRRT similarly indicated amikacin losses of approximately 6–12% [32]. In our data, a time-dependent decline in device-related clearance was not supported, although saturation beyond the sampling period cannot be excluded.

Consistent with these limited observations, a recent systematic review classified amikacin as having no or low removal during CytoSorb^®^ therapy, while emphasizing that the clinical relevance of drug removal depends on patient-, drug-, and circuit-specific factors, including concurrent extracorporeal support [33]. However, these findings may not be directly generalizable to critically ill adults receiving CRRT, and the relative contributions of adsorption, diffusion, and convection remain uncertain. Our study extends this evidence by quantifying the additional device-specific extracorporeal clearance of amikacin in adult septic patients treated with two different extracorporeal modalities. Previous studies have shown that CRRT itself contributes substantially to amikacin elimination. D’Arcy et al. reported a mean amikacin clearance by CVVHDF of 2.86 L/h, with an elimination half-life of 6.74 ± 1.69 h [12], and Roger et al. reported highly variable amikacin clearance by CRRT of 1.8 ± 1.3 L/h during CVVHDF and 1.3 ± 1.0 L/h during CVVH [16]. Our estimates (1.70 and 2.58 L/h) are of a similar order of magnitude to, or modestly higher than, those reported for conventional CRRT; however, because CL_CRRT represents the overall clearance of each extracorporeal configuration, our design cannot attribute any increment to adsorption specifically.

Second, and initially counterintuitively, this pronounced increase in clearance while the device was connected translated into only small differences in simulated day-2 exposure and PK/PD target attainment between configurations and connection timings. Within the simulated scenarios, the dose, rather than the device configuration or the timing of device connection, was the dominant modifiable determinant of target attainment. This apparent tension is explained by exposure kinetics. A configuration that increases clearance by 60–90% while connected accelerates the decline in concentration during that interval; however, Cmax is determined largely by the dose and V1, and AUC24 integrates exposure over the whole day, of which the device was connected for only 10–14 h in our simulations. The net effect on the peak-driven and daily-exposure indices is therefore attenuated, although it may be larger with longer connection times, such as the median treatment durations observed in our cohort (16.0 and 21.75 h). Previous mechanistic guidance suggested administering concentration-dependent aminoglycosides before CytoSorb^®^ initiation or during treatment interruptions to preserve peak concentrations; however, these recommendations were based on limited evidence and did not separate CytoSorb^®^-mediated removal from that attributable to the CRRT platform [34]. Our simulations suggest that fine-tuning the timing of device connection relative to dosing is far less important than ensuring that an adequate dose is prescribed.

Consistent with the findings of Lam et al., amikacin dosing during CRRT should not be determined solely by dialytic dose or conventional bedside variables but rather guided by patient-specific PK evaluation. As optimal antibacterial activity requires a Cmax/MIC ratio of approximately 8–10, a peak concentration of 64 mg/L, corresponding to Cmax/MIC ≥ 8 at an MIC of 8 mg/L, is considered an appropriate target, and achieving it during CRRT may require an initial dose of at least 25 mg/kg [35,36]. Currently used aminoglycoside dosing regimens in critically ill patients receiving CRRT often result in inadequate peak concentrations and poor PK/PD target attainment, with a median administered dose of 14.5 mg/kg [13]. In the present study, target attainment was evaluated using Cmax/MIC ≥ 8 as the primary target [4] and AUC24/MIC ≥ 75 as the secondary target [37]; the latter is derived largely from animal infection models and has not been prospectively validated against clinical outcomes in critically ill patients. Higher AUC/MIC targets have been proposed for infections with a high bacterial burden; however, higher exposure is also associated with an increased risk of nephrotoxicity [38], which should be weighed when targeting values above the threshold used here.

In our simulations, among the regimens tested, only 1500 mg once daily achieved CFR ≥ 90%, and only for the peak-driven C_max_/MIC ≥ 8 target and only against the more susceptible Enterobacterales (Figure 5). The conventional 500 mg once-daily regimen was inadequate across all scenarios, and 500 mg every 8 h, despite an AUC24 similar to that of 1500 mg once daily, achieved lower peak concentrations because the daily dose was fractionated. This dissociation between the two targets is itself instructive: for a concentration-dependent agent whose efficacy is peak-driven while toxicity relates mainly to sustained trough exposure, once-daily high-dose strategy is pharmacodynamically preferable to divided dosing during adsorptive CRRT.

The 1500 mg dose evaluated in our simulations corresponds to approximately 17 mg/kg at the median body weight of our cohort (89 kg), below the loading dose of 25–30 mg/kg that PK/PD simulations suggest is required to achieve adequate target attainment during CVVHDF at MICs ≤ 4 mg/L [39,40]. Consistent with this, 1500 mg once daily maintained ≥90% PTA for Cmax/MIC ≥ 8 only up to an MIC of 2 mg/L; for less susceptible pathogens or heavier patients, weight-based dosing of 25–30 mg/kg should be preferred. In settings where timely TDM and model-informed precision dosing are unavailable, a fixed 1500 mg initial dose may represent a pragmatic option for an average-weight adult, but it should not be considered a universal maintenance regimen. Subsequent dosing should be individualized using the earliest available amikacin concentration, together with body weight, residual renal function, CRRT continuity and microbiological susceptibility, and this simplified strategy requires prospective validation. Although our simulations focused on efficacy, repeated once-daily dosing may lead to accumulation in patients receiving CRRT, whose intrinsic clearance is reduced, and exposure may increase further when the adsorptive device or CRRT is discontinued and total clearance falls by the device-related component, raising the risk of nephrotoxicity and ototoxicity [16]. Subsequent doses and, in particular, dosing intervals should therefore be individualized using TDM, targeting trough concentrations below 2.5 mg/L before the next dose [2,41].

The configuration-specific CL_CRRT estimates should not be interpreted as a comparison of the capacity of the two devices to remove amikacin. The two configurations differ structurally: the oXiris^®^ membrane, an AN69-based polyacrylonitrile membrane, combines filtration with adsorption, whereas in the CytoSorb^®^ configuration, a polymer-bead adsorption cartridge, optimized for mid-molecular-weight mediators, was added downstream of an ST150 hemofilter. Antibiotic adsorption to polyacrylonitrile membranes has been described as material-dependent [15], and experimental data suggest that CytoSorb^®^ hemoadsorption has only modest effects on most anti-infectives, including the aminoglycoside tobramycin [42]; however, because CL_CRRT represents overall extracorporeal clearance, our data cannot distinguish adsorption from diffusive and convective removal. In addition, device selection was non-randomized, the groups were small (11 vs. 13 patients), the SIR-based 95% confidence intervals of the two estimates overlapped substantially, and CL_int showed large between-subject variability (CV 109.6%), so the model may partly attribute unmeasured patient heterogeneity to device type. Although mean baseline renal function did not differ significantly between groups, estimated creatinine clearance was numerically higher and more variable in the oXiris^®^ group (~67 vs. ~42 mL/min), illustrating this risk of confounding. The configuration-specific estimates should therefore be regarded as descriptive rather than as evidence of a difference between devices, which would require a controlled comparative study; moreover, our simulations indicate that, within the evaluated scenarios, the device configuration was clinically less important than the dose.

The magnitude and heterogeneity of CL_int and V1 which we observed (Table 2) are consistent with the well-documented PK instability of aminoglycosides in critical illness, where augmented renal clearance and AKI coexist and can each dominate at different points in a patient’s course. PopPK studies have identified renal function and total body weight as the principal covariates of amikacin clearance and volume of distribution; however, these explain only part of the variability, with IIV in clearance of 28–69% remaining after covariate inclusion [9,43], and high variability has been reported specifically in patients receiving CRRT [16,44]. The IIV in CL_int in our model (CV 109.6%) is in line with this heterogeneity and suggests that relevant patient- and therapy-related determinants remain unidentified. For the same reason, fixed or nomogram-based dosing can provide only an initial strategy in this population: the SIR analysis supported the robustness of the population estimates, but the wide distribution of individual CL_int values indicates that population-average dosing cannot substitute for individual concentration measurements. TDM, ideally combined with model-informed precision dosing, offers a more precise approach by incorporating measured concentrations and patient-specific PK characteristics [41,45].

Phase-imbalanced sampling was present in this study. Rather than over-parameterizing a model the data could not support, we constrained the distribution parameters (Q, V2) with informative priors. This let the richer within-device data inform CL and Vd, while the distributional phase borrowed strength from published amikacin models in comparable populations. The estimates of Q and V2 remained close to the prior means, as intended, and their correlations with the clearance parameters were negligible, indicating that the priors had minimal influence on the clearance estimates; nevertheless, these estimates remain conditional on the assumed distribution model. The cost is visible in the phase-stratified residual analysis, in which the pre-device samples showed greater residual variability than the on-device samples. Nevertheless, CL_int was estimated with acceptable precision (RSE 21.4%) and low η-shrinkage (2.3%), indicating that the combined pre- and on-device data were informative at the individual level. Previous population PK studies have demonstrated that sparse sampling designs provide less information for parameter estimation, resulting in greater uncertainty, reduced precision, and poorer characterization of interindividual variability than designs with multiple observations per subject [46].

Two plausible model extensions were also pre-specified, tested, and rejected. IIV on CL_CRRT was non-identifiable. A single CL_int anchor plus one continuous filter run per patient cannot separate IIV in CL_int from CL_CRRT. Attempting this inflated uncertainty without improving fit. CL_CRRT was therefore retained as a population-level, device-type-specific term. Second, a step-increase in V1 was tested against a reproducible transient decline at device connection (median 21% within an hour, too rapid for elimination alone). It did not improve fit and was imprecisely estimated. A permanent volume change is also the wrong form for a transient event: the increment needed to explain the decline (several liters) far exceeds any plausible circuit-priming volume (typically 150–300 mL). This peri-connection decline is a real but unresolved issue, possibly reflecting acute hemodilution or fluid shifts at CRRT initiation. Confirming its cause would require paired sampling immediately before and after connection, with documentation of concurrent fluids or blood products.

This study has several strengths. It provides one of the first prospective PopPK evaluations of amikacin during CRRT combined with CytoSorb^®^ or oXiris^®^. Its strengths include repeated sampling during active extracorporeal therapy, separate estimation of intrinsic and device-related extracorporeal clearance, model evaluation, and translation of the findings into clinically relevant PTA and CFR simulations.

This study also has several limitations. The sample size was small (24 patients); as a prospective observational study, inclusion reflected real-world practice and the number of eligible patients during the study period, and multiple samples were collected per patient to partially compensate. Device allocation was non-randomized and based on clinical judgement, which may have introduced selection bias, so the configuration-specific CL_CRRT estimates do not support a direct comparison between devices. Pre-device sampling was sparse and no samples were collected after device disconnection; richer pre- and post-device sampling would strengthen the separation of intrinsic and extracorporeal clearance. The estimated CL_CRRT represents overall extracorporeal clearance and cannot distinguish adsorption from diffusive and convective removal. This study was likely underpowered to detect covariate effects; the absence of significant covariates does not exclude clinically relevant effects of established determinants such as renal function or body weight. Because no covariates were retained in the final model, the simulations evaluated fixed doses and could not account for the influence of body weight or renal function on target attainment. The simulations incorporated inter-individual variability but not parameter uncertainty, which may have led to modestly optimistic PTA estimates, particularly for AUC24/MIC and for MICs close to the breakpoint. Overall, our findings should be regarded as exploratory and require confirmation in larger, prospective studies.

## 5. Conclusions

In critically ill septic patients, CRRT combined with CytoSorb^®^ or oXiris^®^ was associated with a substantial increase in amikacin clearance while the device was connected. However, within the evaluated simulation scenarios, target attainment was influenced more strongly by the dosing regimen than by device type or the timing of device connection. Among the regimens tested, 1500 mg once daily provided the highest probability of achieving the PK/PD target, although adequate target attainment was limited to the more susceptible Enterobacterales. These findings support an adequate weight-based initial dose, followed by early TDM and individualized redosing. Where timely TDM is unavailable, a fixed 1500 mg initial dose may represent a pragmatic option for an average-weight adult, but subsequent dosing should be individualized as soon as clinical and concentration data become available. Given the small, non-randomized design, these findings, including the configuration-specific clearance estimates, should be considered exploratory and require prospective external validation.

## Figures and Tables

**Table 1:** Baseline patient characteristics and dataset composition.

Characteristic, Units	Median (Interquartile Range) [Range]/ Number (%) (N = 24)
Age, years	60.5 (52.2–69.0) [27–88]
Weight, kg	89.0 (87.8–93.2) [63–130]
Height, cm	182.5 (171.8–185.0) [160–193]
Body mass index, kg/m^2^	27.6 (26.0–30.3) [21.8–39.2]
Sex, male/female	17 (70.83)/7 (29.17)
Serum creatinine, µmol/L	278 (117–372) [39–1208]
Estimated creatinine clearance, mL/min (before treatment)	31.1 (20.5–78.5) [6.6–211.9]
Serum albumin, g/L	28.5 (24.0–31.0) [18–35]
C-reactive protein, mg/L	270 (119–344) [29–615]
Interleukin-6, pg/mL	587 (155–1835) [3.7–50,000]
Extracorporeal filter, CytoSorb^®^/oXiris^®^	11 (45.83)/13 (54.17)
Concentrations analyzed pre-CRRT_CRRT/CRRT(CytoSorb)/CRRT(oXiris)	22 (14.10)/61 (39.10)/73 (46.80)

**Table 2:** Final population pharmacokinetic amikacin model parameter estimates.

Parameter (Units)	Estimate	RSE (%)	SIR Median [95% CI]
Intercompartmental clearance, Q (L/h) ^a^	4.70	13.3	4.74 [3.58–5.93]
Peripheral volume, V2 (L) ^a^	9.15	8.7	9.18 [7.62–10.56]
Intrinsic clearance, CL_int (L/h)	2.79	21.4	2.74 [1.44–3.97]
Central volume of distribution, V1 (L)	19.3	12.5	19.2 [13.7–24.0]
Extracorporeal clearance during CytoSorb, CL_CRRT(CytoSorb) (L/h)	1.70	26.8	1.74 [0.86–2.57]
Extracorporeal clearance during oXiris, CL_CRRT(oXiris) (L/h)	2.58	21.2	2.65 [1.67–3.69]
Proportional error (%)	18.6	16.4	18.3 [11.3–23.8]
Additive error (mg/L)	1.57	19.2	1.56 [1.04–2.17]
IIV CL (CV%) ^b^	109.6	18.9	115.8 [58.7–175.4]
IIV V1 (CV%) ^b^	45.1	23.0	47.7 [26.3–65.2]
Covariance CL–V1	0.318 ^c^	36.7	0.334 [0.090–0.550]
η-shrinkage, CL/V1 (%)	2.3/8.2	-	-
ε-shrinkage (%)	9.8	-	-

^a^ Estimated with informative priors (NWPRI). ^b^ $CV\%=\sqrt{exp(\omega^{2})-1}*100$; ^c^ corresponding correlation coefficient (r) = 0.83; SIR CIs were derived by transforming the ω^2^ percentiles. CI, confidence interval; CV, coefficient of variation; IIV, interindividual variability; RSE, relative standard error; SIR, sampling importance resampling.

## Data Availability

The datasets used and analyzed during the current study are not publicly available owing to ongoing studies but are available from the corresponding author upon reasonable request.

## Abbreviations

The following abbreviations are used in this manuscript:

CL_int	intrinsic clearance
CL_CRRT	extracorporeal clearance
pcVPC	prediction-corrected visual predictive checks
AKI	acute kidney injury
SA-AKI	sepsis-associated acute kidney injury
PTA	probability of target attainment
CFR	cumulative fraction of response
CL_TOT	total clearance
PK	pharmacokinetics
CRRT	continuous renal replacement therapy
SIR	sampling-importance-resampling
TDM	therapeutic drug monitoring
PopPK	population pharmacokinetic
CVVHDF	continuous veno-venous hemodiafiltration
SCr	serum creatinine
Alb	serum albumin
CRP	C-reactive protein
IL-6	interleukin-6
V1	central volume of distribution
IIV	interindividual variability
SCM	stepwise-covariate-modeling
OFV	objective function value
PRED/IPRED	population/individual predictions
CWRES	conditional weighted residuals
GOF	goodness-of-fit
CI	confidence interval
V2	peripheral volume of distribution
